# Large cognitive fluctuations surrounding sleep in daily living

**DOI:** 10.1016/j.isci.2021.102159

**Published:** 2021-02-07

**Authors:** Reto Huber, Arko Ghosh

**Affiliations:** 1 Child Development Center, University Children's Hospital Zurich, Switzerland & Department of Child and Adolescent Psychiatry and Psychotherapy, Psychiatric Hospital University of Zurich, Switzerland; 2 Institute of Psychology, Cognitive Psychology Unit, Leiden University, Wassenaarseweg 52, Leiden 2333 AK, the Netherlands

**Keywords:** Biological Sciences, Neuroscience, Behavioral Neuroscience, Cognitive Neuroscience

## Abstract

Cognitive output and physical activity levels fluctuate surrounding sleep. The ubiquitous digitization of behavior via smartphones is a promising avenue for addressing how these fluctuations occur in daily living. Here, we logged smartphone touchscreen interactions to proxy cognitive fluctuations and contrasted these to physical activity patterns logged on wrist-worn actigraphy. We found that both cognitive and physical activities were dominated by diurnal (∼24 h) and infra-radian (∼7 days) rhythms. The proxy measures of cognitive performance—tapping speed, unlocking speed, and app locating speed—contained lower-powered diurnal rhythm than physical activity. The difference between cognitive and physical activity was vivid during bedtime as people continued to interact with their smartphones at physical rest. The cognitive performance measures in this period were worse than those in the hour before or after bedtime. We suggest that the rhythms underlying cognitive activity in the real world are distinct from those underlying physical activity, and this discord may be a hallmark of modern human behavior.

## Highlights

• Daily and weekly rhythms shape our day-to-day behavior
• The speed of smartphone interactions fluctuates according to the time of the day
• These fluctuations do not strictly follow the physical activity cycles
• The worst performing time on the smartphone is around bedtime

## Graphical abstract

**Figure:**
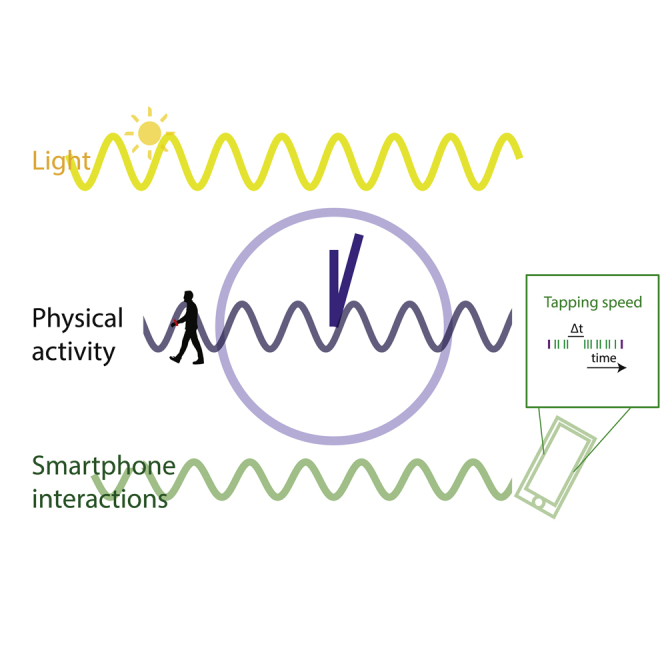

## Introduction

Cognitive performance systematically fluctuates according to rhythms of different lengths. The best-studied rhythms in a laboratory setting are diurnal (Dijk et al., 1992; Wright et al., 2012). Cognitive rhythms have also been explored in the context of seasonality, menstrual cycle, weekly cycle, and cycle with a 90-min period (inspired by basic rest-activity) (Huttenlocher et al., 1992; Klein and Armitage, 1979; Kleitman, 1982; Lavie, 1980; Sommer, 1973). Establishing such rhythms is a key step towards the mechanisms underlying systematic behavioral variations. For instance, whereas the diurnal cycle may be attributed to intrinsic circadian clocks, the weekly cycles may be attributed to the artificial schedules of human society. The mechanistic understanding also hinges on whether the rhythms appear systemwide or if they are compartmentalized. For instance, the circadian rhythmicity and the underlying clocks vary across different brain areas, and between the suprachiasmatic nucleus (the “master clock” in the brain) and the muscles (Abe et al., 2002; Gabriel and Zierath, 2019). These observations in laboratory settings raise the question of whether in daily living certain rhythms dominate human behavioral outputs and if their influence is compartmentalized to specific domains.

The endogenous circadian rhythm significantly impacts various cognitive performance measures in a ∼24-h rhythm (Borbély et al., 2016; Schmidt et al., 2007). In addition, cognitive performance suffers under high sleep pressure as a function of the duration of prior wakefulness (Borbély et al., 2016; Lo et al., 2016). According to a well-established idea, the interaction between the circadian timing and time awake modulates cognitive performance (Dijk et al., 1992). To elaborate, the circadian timing impacts the steady deterioration of performance with increasing wake duration both in a favorable and in an unfavorable way. This impact is best seen during the so-called wake maintenance zone, a time window 2–3 h before the start of melatonin secretion in the evening, during which the circadian system maximally supports wakefulness and cognitive performance (Lo et al., 2012). On the other hand, a trough in circadian support is seen during nighttime supporting consolidated sleep (Zhou et al., 2011). Additionally, sleep inertia, i.e., morning grogginess, reduces performance in the first 30–90 min after waking up (Wertz et al., 2006).

In laboratory settings the baseline measures before sleep deprivation offer some key insights into the compartmentalized nature of diurnal cognitive rhythms (Blatter and Cajochen, 2007; Burke et al., 2015). First, sensorimotor cognition captured by using a psychomotor vigilance performance test remains stable through the waking hours before the sleep deprivation, but memory performance captured using a different test fluctuates through the day in the same period (Chua et al., 2014; Schmidt et al., 2007). Second, if people are woken up from their sleep to perform tasks akin to emergency response by a medical worker, executive functions, in particular, appear vulnerable (Horne and Moseley, 2011).

Wearables with accelerometers (actigraphy) are widely used in the study of rhythms that dominate daily living, albeit with a focus on overall physical activity rather than cognition and on the diurnal rhythm rather than infra- or ultra-radian rhythm. Still, diurnal, infra- and ultra-radian rhythms are all visible in the movements logged using actigraphy (Fossion et al., 2017; Wong et al., 2013). Of interest, the inter-individual differences in the near-24-h rhythm of physical activity may be markers of clinical conditions (Germain and Kupfer, 2008; Krafty et al., 2019; Leng et al., 2020). The observation in daily living that diurnal rhythms for the left versus right arm differ further support the idea that these rhythms can be compartmentalized in the nervous system (Natale, 2002).

One promising avenue to study the cognitive oscillations in the real world is to leverage the daily digital interactions and yield proxy measures of cognitive functions (Balerna and Ghosh, 2018; Insel, 2017; White and Horvitz, 2019; Austin et al., 2011). According to one recent report leveraging the keypresses while on a web search engine, the speed of the keypresses fluctuates according to the time of the day, similar to what has been found for reaction time tasks in the laboratory (Althoff et al., 2017). Smartphone touchscreen interactions log (tappigraphy) is particularly suitable for long-term assessments spanning virtually all the waking hours. Indeed, the usage occupies the waking hours such that the distribution of the smartphone touchscreen interactions can proxy sleep-wake times (Borger et al., 2019; Min et al., 2014). Moreover, the smartphone can also sample behavior while lying in bed putatively awaiting sleep or recovering from sleep inertia (Borger et al., 2019).

The combination of actigraphy and tappigraphy in the same individual can help address whether the rhythms in cognitive activity differ from that of physical activity in the real world. Can different cognitive domains be evaluated based on smartphone interactions? There is emerging evidence that this may indeed be possible. Inter-individual differences in smartphone tapping speeds (TSs) are strongly correlated to motor variability in tactile reaction time tasks and weakly correlated to the reaction time (Balerna and Ghosh, 2018). The same parameter is strongly correlated to 4-choice reaction times in response to visual stimuli and weekly correlated to simple reaction times (Akeret et al., 2020). This pattern of results suggests that TS can be used to proxy executive functions (Stuss et al., 2003). Furthermore, another smartphone parameter—unlocking speed (US)—is unrelated to reaction time performance (Akeret et al., 2020). In sum, distinct smartphone parameters can help address the domain-specific variations in cognitive rhythms.

In this study we used the following different tappigraphy parameters to proxy cognitive processes: (1) TS, the time that is taken to go from one touch to another; (2) US, the time that is taken to unlock the phone; and (3) app locating speed (ALS), the time that is taken to locate app icons on the home screen before launch - inspired by conventional visual search tasks (Wang et al., 1994). These yielded time series of measurements enabling spectral density analysis of the cognitive fluctuations to identify the oscillations that dominate the cognitive outputs, with periods of ∼24 h and larger. We contrasted these measures to actigraphy (including ambient luminescence) captured using a wrist-worn wearable. Finally, we tethered our analysis to actigraphy-estimated sleep times to capture the putative impact of sleep inertia and pressure across the different cognitive domains captured using tappigraphy.

## Results

### Periodicity in luminescence, physical activity, and tappigraphy

**Figure 1:**
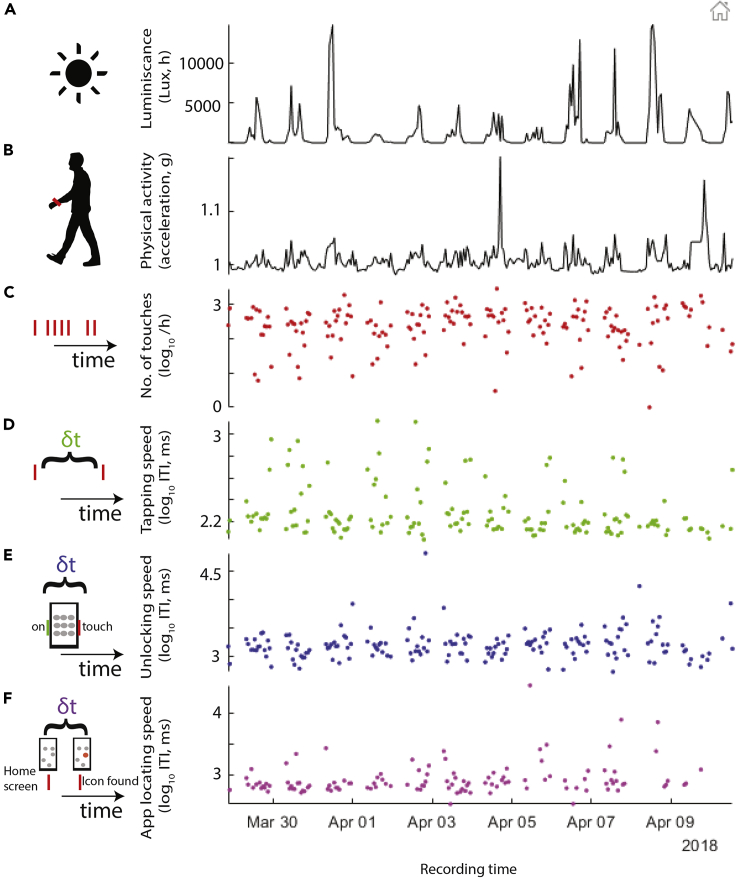
The range of measures captured in daily living conditions in this study (A and B) Wearable and smartphone data from one exemplary subject monitored over 21 days. (A) The amount of ambient light was captured on the wrist along with (B) the acceleration used here as a proxy for physical activity. (C–F) We quantified the smartphone behavior in hour-long bins in terms of (C) the number of touches, (D) the speed of the interactions measured as the interval between subsequent touches (fastest 25^th^ percentile at each bin, tapping speed, TS), (E) the time taken to unlock the screen (unlocking speed, US), and (F) the time to select an app icon while on the home screen (app locating speed, ALS).

**Figure 2:**
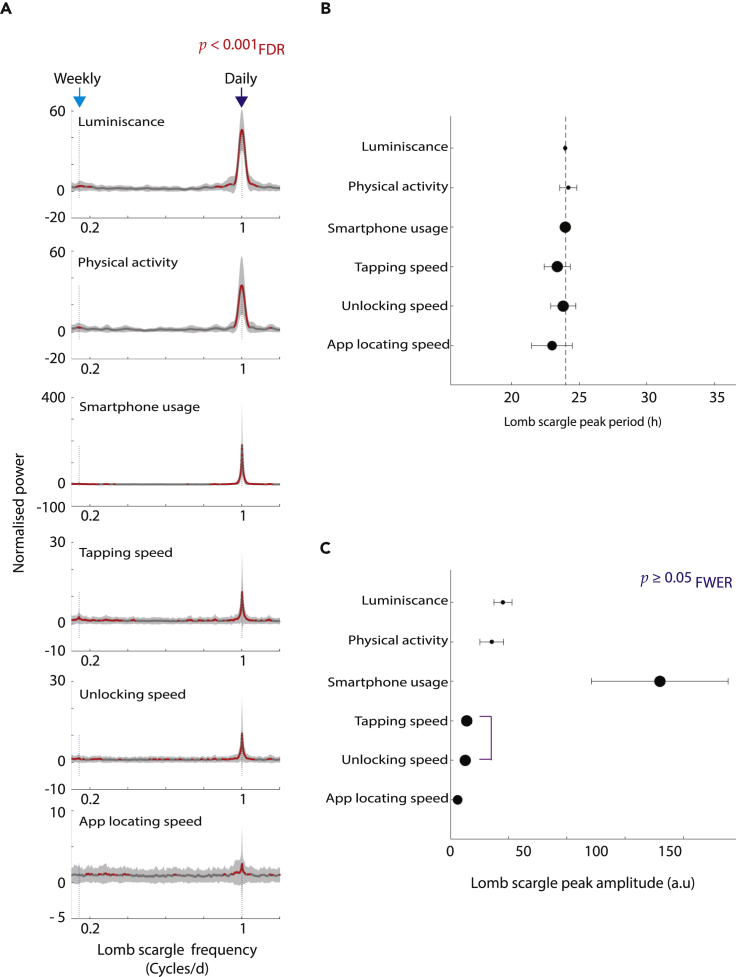
Lomb-Scargle periodogram reveals the periods that dominate the wearable (luminescence and physical activity) and smartphone parameters (A) Mean periodograms and their corresponding confidence intervals (95%), with significant differences from zero-amplitude signal marked in red. (B) Mean peak periods derived from the periodograms and their corresponding confidence intervals. (C) Mean peak normalized powers and their corresponding confidence intervals. The sizes of the shapes in (B and C) correspond to the sample size (see main text for the corresponding degrees of freedom).

The wearable and tappigraphy signals fluctuated through the recording period (Figure 1). To quantify the periodicity of these fluctuations, we used the Lomb-Scargle method. Population average traces were used to establish the consistency of the behavioral patterns in the sampled population controlled for multiple comparisons by using false discovery rate, FDR (Pernet et al., 2011). The periodograms revealed a consistent ∼24-h periodicity across all the signals and a less prominent ∼7-d periodicity for the ambient luminescence, physical activity, smartphone usage (number of touches), and the two proxy measures of cognitive processing speed, TS and US (Figure 2A).

By using the periodogram peaks we next estimated which period consistently dominates the signals (Figure 2B). First, we contrasted the location of the periodogram peaks with 24-h periods using one-sample t tests to establish deviations from this anticipated period (α_FWER_ = 0.0083, Benjamini and Yekutieli, 2001). The mean peak periodicity of ambient light fluctuations was 23.96 (p = 0.002, *t*(69) = −3.27), for movements it was 24.19 (p = 0.22, *t*(70) = 1.23), for smartphone usage it was 23.97 (p = 4.24 x 10 ^−6^, *t*(183) = −4.74), for TS it was 23.38 (p = 0.01, *t*(184) = −2.57), for US it was 23.82 (p = 0.43, *t*(184) = −0.78), and for ALS it was 23.00 (p = 0.01, *t*(156) = - 2.65). Next, we compared the peak locations across the signals to find that the periods were domain dependent (p = 0.01, *f*(5,846) = 3.01, ANOVA).

There were pronounced inter-parameter differences between the power estimates (i.e., in the normalized peak amplitudes of the periodogram, p = 7.22 x 10^−74^, *f*(5,848) = 87.02, ANOVA, Figure 2C). The power of smartphone usage showed the highest ∼24-h peaks relative to any of the signals, and the ALS showed the weakest peaks. Notably, the proxy measures of cognitive processing (TS, US, and ALS) speed show a lower amplitude than the wearable measures of luminescence or physical activity (Figure 2C).

We performed an additional analysis based on tappigraphy parameters accumulated at 15-min bins to address the putative presence of cognitive rhythm with a 90-min period. Although this form of accumulation again revealed strong diurnal cycles, none of the tappigraphy parameters revealed a consistent 90-min period (Figure S1).

### Time-of-the-day effects in physical and cognitive signals

**Figure 3:**
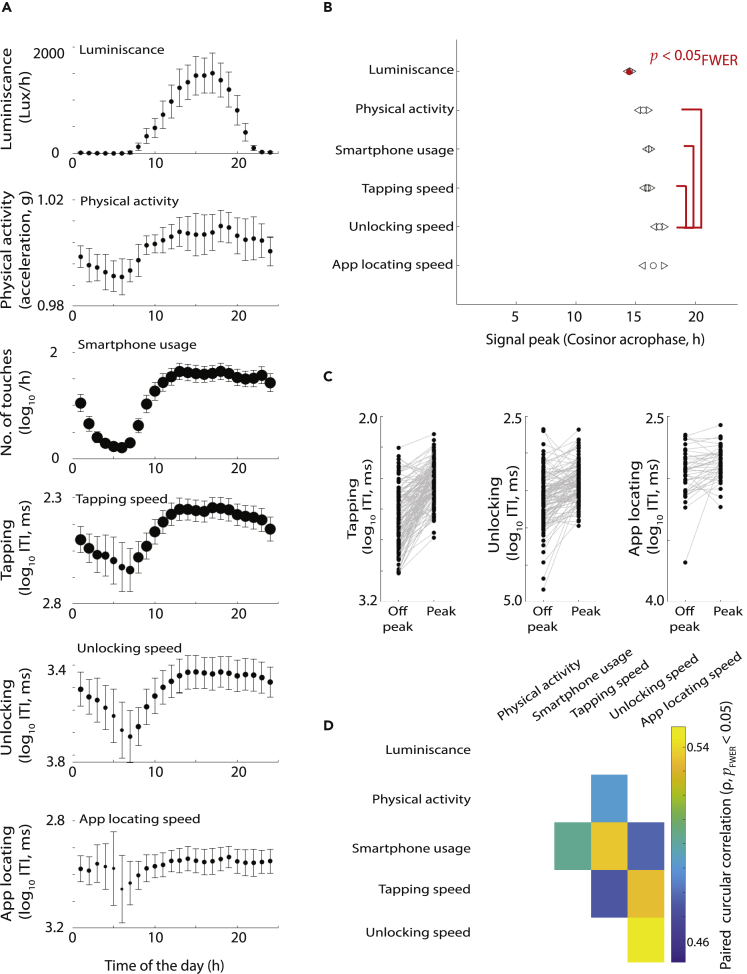
Time of the day reflects on physical activity and processing speeds captured on the smartphone (A) Time-of-the-day fluctuations in mean values and the corresponding 95% confidence intervals. (B) Cosinor fits revealed the period of signal peak (higher amplitude of movements, luminescence, and smartphone usage and smaller inter-touch intervals for processing times) on the 24-h clock, with the confidence intervals marked with triangles. (C) The comparison of processing at the acrophase versus off phase (bathyphase) in the sample, with each individual represented with a connecting line. (D) The inter-individual differences in the time of peak performance (cosinor, acrophase) are related to each other. The circular correlation coefficient is shown for the significant relationships.

Upon establishing the dominance of ∼24-h rhythms in the physical and cognitive fluctuations we next focused on how the gathered measures related to the time of the day. As anticipated from the periodograms, the central tendencies revealed substantial time-of-the-day fluctuations across the parameters (Figure 3A). To systematically address at which hour the signals consistently peaked we relied on cosinor analysis with a fixed 24-h waveform (with individual fits corresponding to p < 0.05, Nelson et al., 1979). The hour at which performance peaked (the cosinor acrophase) depended on the parameter (p = 0, *f*(5,673) = 25.64, Watson-Williams multi-sample test, Berens, 2009). Follow-up tests revealed that the peak for ambient light exposure preceded all other measures, whereas the peak for US lagged all other measures except for ALS (Figure 3B). Although the hour of peak performance for the proxy measures of cognitive processing occurred between 16 and 17 h, the differences between the peak and off-peak performance was the most pronounced for TS (p = 3.23 x 10^−29^, *t*(125) = 14.78, paired t test), followed by US (p = 3.48 x 10^−12^, *t*(117) = 7.76) and the ALS (p = 0.0062, *t*(39) = 2.89) (Figure 3C).

Next, we addressed whether the inter-individual differences in the acrophase were correlated across parameters (Figure 3D). In particular, we were interested in the putative determinants of the cognitive processing proxied here. We used paired circular correlations to address these relationships. Of interest, the subtle variations in the ambient light acrophase were not correlated to any of the measures. However, physical activity was correlated to only one of the tappigraphy measures: US. Furthermore, smartphone usage was related to all the proxy measures of processing speed.

The analysis presented above is based on 24-h sinusoids, and in notable proportion of the data a corresponding rhythm could be detected (the null hypothesis of no rhythm could not be eliminated for 0% of the sampled population for luminescence, 1% for physical activity, 1% for smartphone usage, 18% for TS, 20% for US, and 70% for ALS). We performed an additional set of analysis to estimate the signal peaks, and thus untethered our analysis from the sinusoid. This analysis confirmed that the high-performing (larger signal amplitude) time-of-the-day hour bins were dependent on the parameter (p = 0.0011, *f*(5,556) = 4.14, Watson-Williams multi-sample test), with luminescence preceding physical activity, TS, and US (Figure S2).

Similarly, we also followed up on the ∼7-day rhythm identified using the periodogram based on time-of-the week analysis to find systematic variance according to the day of the week for all measures except the US and ALS (Figure S3). The day on which the signals peaked varied according to the measured parameter (p = 0, *f*(5,838) = 52.77, Watson-Williams multi-sample test). Furthermore, whereas physical activity and luminescence peaked around the weekend, smartphone usage and TS peaked around the weekday.

### Poor cognitive performance during actigraphy-labeled “sleep”

**Figure 4:**
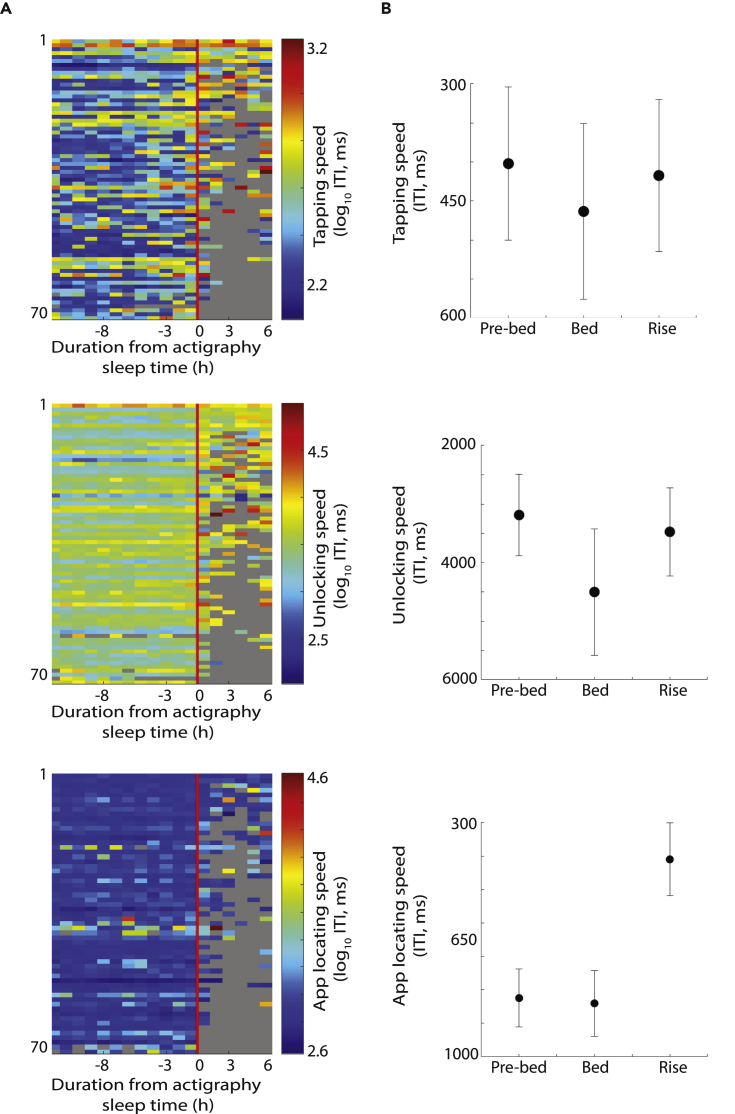
Cognitive processing speed captured on the smartphone during actigraphy-labeled sleep (A) The median processing speeds—in terms of tapping speed, unlocking time, and locating the app icon—captured for each individual accumulated over the observation period. (B) The differences in mean processing speed captured during actigraphy-estimated sleep in contrast to the values accumulated in the hour before sleep and after sleep (95% confidence interval). The sizes of the shapes in (B) correspond to the sample size (see the main text for the corresponding degrees of freedom).

The prominent diurnal cycles in cognitive rhythms may be partly due to sleep-related influences on cognitive performance. To explore this we focused on the sleep-wake transitions and, more uniquely, while in actigraphy-defined sleep (i.e., while lying still in bed and using the smartphone and yet classified as sleep by the Cole-Kripke algorithm on actigraphy, Cole et al., 1992) (Figure 4A). From each individual, we pooled all the instances of usage at three different periods: *pre-bed* defined as 1 h immediately preceding actigraphy-defined sleep time, *bed* defined as during the putative sleep (actigraphy defined) time, and *rise* defined as 1 h immediately following the sleep period (Figure 4B). We focused on the subset of the population where the parameter estimation requirements were satisfied to yield a measure in each of these periods (Figure 4B). TS fluctuated only marginally across the three periods (p = 0.07, *f*(2,65) = 2.73, ANOVA). Follow-up paired t tests revealed marginal slowing in *bed* versus *pre-bed* (p = 0.05, t(65) = −2.0) and the *bed* versus *rise* (p = 0.05, *t*(65) = 2.04). Furthermore, there was no difference between the *pre-bed* versus *rise* (p = 0.59, *t*(65) = −0.54). US fluctuated substantially through these periods (p = 5.62 x 10-9, *f*(2,62) = 22.23), and the follow-up t tests revealed a similar pattern as for TS albeit more exaggerated. The *bed* period compared with the *pre-bed* was substantially slower (p = 5.01 x 10^−7^, *t*(62) = −5.6), and the *bed* period was also slower versus the *rise* period (p = 6.12 x 10^−5^, *t*(62) = 4.3). There was a marginal difference between the *pre-bed* and *rise* periods, with the *rise* being slower (p = 0.03, *t*(62) =-2.20). ALS too fluctuated through these periods (p = 5.50 x 10^−30^, *f*(2,47) = 150.08). Interestingly, the *bed* period was no different versus the *pre-bed* period (p = 0.52, *t*(47) = −0.64). However, the *rise* period was faster than both *pre-bed* (p = 0, *t*(47) = 12.98) and *bed* periods (p = 0, *t*(47) = 15.53).

Intuitively, these distinct performance levels around sleep may be influenced by the day of the week. For instance, the urgency of actions performed through the weekend may be distinct from the weekday. To address this, we separated all the sleep-related measures into two sets depending on the sleep onset: (1) spanning Friday, Saturday, and Sunday and (2) spanning Monday, Tuesday, Wednesday, and Thursday. The same pattern of results for *pre-bed*, *bed,* and *rise* times was obtained across these two sets (Figure S4).

## Discussion

The proxy measures of cognitive processes captured using tappigraphy revealed a range of systematic fluctuations, and here we contrasted these to the fluctuations in ambient luminescence and physical activity. The tappigraphy measures—like luminescence and physical activity—were dominated by ∼ 24-h rhythm. However, there were visible differences between the cognitive and the non-cognitive rhythms in terms of the exact period, the power, and even the time of the day when they peak. Some of these differences also extended to the ∼7-d rhythm. Intriguingly, the tappigraphy measures also allowed us to assess the performance at odd hours, including when in bed, revealing a distinctly slow cognitive output.

As continuous measures related to cognitive output are mostly unexplored, the general patterns of the signals observed in tappigraphy are of interest. The periodograms of the three proxy measures of cognitive processing speed (TS, US, and ALS) revealed dominant powers at frequencies with periods of ∼24 h and ∼7 d. Although all the measures were dominated by 24-h rhythms, there were substantial inter-individual differences and the exact period varied according to the parameter. This is in line with previous observations demonstrating deviations from the 24-h period in cognitively rooted performance parameters such as handgrip strength (Ashkenazi et al., 1993). We also observed ∼7-d rhythm in the cognitive parameters of smartphone usage and TS. This supports the idea that the time information—both in terms of time of the day and time of the week—is encoded in cognitive output (Huttenlocher et al., 1992).

We anticipated ∼7-d, 24-h, and 90-min rhythms in tappigraphy. We did find evidence for the first two, whereas we found no indications of the third rhythm. Ninety-minute rhythms play an important role in sleep, but their impact on wakeful periods and cognition is contentious. The original observations of 90-min fluctuations in cognitive tests have been difficult to reproduce and attributed to low-threshold statistical tools (Klein and Armitage, 1979; Neubauer and Freudenthaler, 1995).

Luminescence is a primary *zeitgeber* for circadian physiological rhythms according to observations mostly in the sleep laboratory (Bedrosian and Nelson, 2017; Chellappa et al., 2011; Wever, 1989). Still, cognitive processes may not be faithfully tethered to this in the real world. First, the cognitive processing measures were less dominated by ∼24-h rhythms when compared with the luminescence captured at the wrist. Second, according to the fixed (24-h) cosinor analysis, all the proxy measures of cognitive processing peaked (became faster) later in the day when compared with the experienced luminescence. Finally, population-level variance in tappigraphy time to peak was unrelated to the variance in the experienced luminescence. We speculate that cognitive processes follow diurnal rhythms that are partially independent of the experienced luminescence. Nevertheless, as the luminosity sensor on the wrist was insensitive to the light emitted from the smartphone itself and as the cosinor peaks corresponding to the proxy measures of cognitive performance were related to smartphone usage, it is possible that the cognitive processes track the smartphone-emitted light as opposed to natural light.

The processes that drive the well-documented rhythmicity in overall physical activity may not entirely overlap with the oscillators underlying cognitive activity in the real world. Toward this, cosinor analysis revealed some important separations between cognitive and physical activity. The US peaked later than physical activity, and the variations in time to peak in physical activity were related to only one of the tappigraphy measures—US. The differences between physical and cognitive activity were further widened in the ∼7-d rhythms, and whereas tappigraphy measures (smartphone usage and TS) peaked during weekdays, physical activity (and experienced luminescence) peaked during weekends.

Of interest, smartphone behavior showed the strongest ∼24-h power (signal normalized) when compared with the other measures considered here. This underscores the habitual nature of smartphone behavior where it may be more driven by daily rhythm than overall physical activity or the amount of light exposure. Nevertheless, the daily rhythms were less powerful for the proxy measures of cognitive output, suggesting discord between engaging in smartphone behavior and the cognitive processing speed proxied on the smartphone. This discord was evident in the time-of-the-day analysis, and by using the fixed 24-h cosinor analysis we found that the US peaked (was the fastest) later in the day when compared with smartphone usage. This raises the possibility that the need or desire to engage on the smartphone and certain cognitive performance abilities is out of sync, i.e., phase shifted.

In contrast to diurnal rhythms, the ∼7-d (circaseptan) rhythms in human physiology and behavior have received less attention. We observed this rhythm for smartphone usage and TS. Although intuitively they may stem from the widespread use of the weekly calendar, there is notable evidence suggesting a more intrinsic biological substrate (Cornélissen et al., 2000). Cognitive processing as captured in reaction time tests also shows ∼7-d rhythms (Beau et al., 1999). Perhaps the slow progress in understanding this domain is partly linked to the methodological obstacles. The observation of Beau et al. (1999) still holds today for conventional tests: “The difficulties inherent in such a study is numerous, including … the need to administer tests every day of the week.” The approach used here helps overcome this key barrier.

By leveraging smartphone touchscreen behavior, we could sample cognitive fluctuations at the gray zone between sleep and wakefulness. People spontaneously interacted with their smartphones in the actigraphy-labeled sleep periods, and we leveraged these interactions to address the cognitive status in this “sleep” period when compared with the performance 1 h before and after this period. Now, admittedly actigraphy can overestimate sleep and people may engage on their smartphones while at rest in bed (Borger et al., 2019). Still, this provided us with an opportunity to assess cognition in this period of sleep fracture. Across the different proxy measures of cognitive processing, the performance was poor in this obscure period. The mechanism underlying this, as in sleep inertia versus pressure, could not be clarified without polysomnography; it is possible that the participants intermittently woke up from sleep in the bed (inducing inertia) and it is equally possible that they may have remained still without sleep (building sleep pressure). The current pattern suggests a dual contribution. In the hour after sleep, inertia can be considered to be maximal and yet the performance at sleep fracture was worse than this period. This suggests that an additional factor, such as sleep pressure, is compounded with sleep inertia to additionally degrade cognitive output in the obscure sleep period. Conversely, in the hour before sleep, sleep pressure can be considered to be maximal, and yet TS and US degraded further at sleep fracture. Interestingly, ALS did not degrade further and perhaps the underlying processes are particularly sensitive to pressure rather than inertia. Such specific variations or lack thereof are in keeping with the general notion that sleep impacts cognitive processes in a domain-specific manner (Burke et al., 2015; Ferrara et al., 2000). Nevertheless, factors besides sleep inertia and pressure may have contributed to our results. Under sleep deprivation, altering body posture alone (from standing to sitting) is known to impact cognitive performance (Caldwell et al., 2003).

### Limitations of the study

Our approach of assessing cognitive fluctuations surrounding sleep in daily living conditions requires further consideration. First, we observed different rhythms in cognitive versus the physical activity measures, including the presumable *zeitgeber* of ambient light. It was not clear if these asynchronies were introduced by smartphone behavior or if they are intrinsic properties captured on the smartphone. On a related note, the consequences of the differences between smartphone usage and the proxy measures of cognitive processing too need further exploration. These asynchronies may have important consequences for mental and physical well-being (Van Someren and Riemersma-Van Der Lek, 2007). Second, there is much to be addressed on why and how people behave at physical rest while in bed (i.e., actigraphy-defined sleep). What are the cognitive and behavioral processes underlying these behaviors that spontaneously occur so close to sleep? Perhaps the gold-standard measure of sleep will help better understand the bedtime sleep-cognition interactions better. Third, we deployed Lomb-Scargle spectrograms and cosinor analysis to capture the rhythms. Both of these approaches are extensively used in basic and clinical research (Cornelissen, 2014; Ruf, 1999). However, there are emerging alternatives that may be more sensitive to detecting subtle rhythms (Fossion et al., 2017). We anticipate that the data shared with this report will further help the development of such alternative methods. Fourth, our study population was dominated by students. How these findings extend to the general population remains to be seen. Finally, unlike traditional cognitive testing, the parameters extracted from spontaneous smartphone behavior do not allow us to simply specify the cognitive processes with precision even if they are highly correlated to conventional reaction time. For instance, the TS may reflect not only the underlying cognitive process but also the momentary behavioral demands, as in typing and urgent message versus relaxed web browsing. At least in measures such as the ALS and US the range of actions was more constrained. Meaningful cognitive processes are inherently complicated, and the approach of tappigraphy and the findings presented here is a key step to help unravel that complexity.

### Resource availability

#### Lead contact

Arko Ghosh, Leiden University, The Netherlands.

#### Materials availability

Not applicable as no specific reagents were used for this study.

#### Data and code availability

The data used in this study—from smartphones and wearables—are made available at dataverse.nl, (https://doi.org/10.34894/6CIGDY) along with the codes to analyze the data.

## Methods

All methods can be found in the accompanying Transparent Methods supplemental file.

## Supplemental information

Document S1. Transparent methods and figures S1–S5
