# Supplementary material for: Large cognitive fluctuations surrounding sleep in daily living
Source: iScience. 2021 Feb 7;24(3):102159. doi: 10.1016/j.isci.2021.102159 (PMC7918275; doi:10.1016/j.isci.2021.102159)
Supplement: Document S1. Transparent methods and figures S1–S5 [file mmc1.pdf]

iScience, Volume *24*

Supplemental information

Large cognitive fluctuations surrounding sleep in daily living

Reto Huber and Arko Ghosh

Transparent Methods

Participants

A total of 189 individuals were sufficiently sampled (with 235 addressing the recruitment call). To be included in the study the subjects self-reported that they were healthy and without any ongoing neurological disease or medication. The data collection and analysis were approved by the ethical committees of Leiden University (Psychology Research Ethics Committee) and the medical ethics committee of Arnhem-Nijmegen. All subjects provided informed consent for the study. The age (reported by 164 participants) was a median of 22.4 years (min, 16.1 and max, 45.1) at the time of study consent. The sex (reported by 132 participants) was 70 females and 62 males. The primary occupation was reported by 118 participants, and 89 of them reported being a student. None of the reported professions required night shift work.

Actigraphy measurement

Actigraphy measures were obtained from a subset of participants (n = 79) and reported in a previous study (Borger et al., 2019). Participants wore GENEACTIV watches (Activinsights, Cambridgeshire, UK) on both the wrists, but only the measures from the left wrist were used here. The watches measured the 3-axis accelerometry along with the ambient luminesce and near body temperature, but only the former two measures were used here. The ambient luminesce sensor was insensitive to the light emitted from the smartphone (Suppl. Fig. 4). The participants were instructed to wear the watches for 3 weeks continuously and this yielded measure lasting for a median of 21 days (min, 7 and max, 32). The 3 axis accelerometry was reduced by using $M = \sqrt{(x^2 + y^2 + z^2)}$, where M is the value used here and x, y and z correspond to the accelerations on the distinct axis. The Cole–Kripke

algorithm was used to label sleep periods based on these measures as described in detail elsewhere along with the corresponding MATLAB codes (Borger et al., 2019; Cole et al., 1992).

Smartphone measurements

The timestamp of touchscreen interactions and the corresponding app labels (as in Facebook, Launcher screen, Weather) were recorded using an app running in the background of the user's device (TapCounter, QuantActions, Lausanne, Switzerland). The app required an Android operating system. Based on this labelled time-series of events the following parameters were estimated in hourly bins: (a) Smartphone usage, in the form of number of touchscreen interactions in each bin while the phone was in an unlocked state, (b) tapping speed, in the form of the 25th percentile inter-touch interval accumulated from all of the screen ON sessions in each bin, (c) unlocking speed, in the form of 25th percentile inter-event interval between the two intervals, one, the touchscreen turning ON and two, the touch on the unlocked screen and, (d) app locating speed, as in the inter-touch interval between two consecutive touches on the home/launch screen (identified using the corresponding app label) before the launching of any app. As with the previous measures the 25th percentile of the intervals in each hour bin was recorded. All of the smartphone parameters were transformed by using log_{10}.

The rationale behind the smartphone proxies of cognitive performance

The three measures of cognitive performance were inspired by conventional measures of cognitive performance, and all of them share the property of overcoming the bounds of conventional measurements constrained by the laboratory setting or task. (a) Tapping speed:

The finger-tapping task is commonly used to assess motor speed and is highly related to the inter-keystroke intervals on a keyboard (Austin et al., 2011). However, the inter-touchscreen intervals offered a crucial advantage as the smartphone interactions are likely to occur more spontaneously and even in bed in contrast to the interactions on the personal computer. This measure is related to tactile reaction times, visual reaction times and tactile reaction time variability (Akeret et al., 2020; Balerna and Ghosh, 2018). (b) Unlocking speed: This measure captures the memory dependent cognitive processes. Moreover, the time taken to perform this task is expected to be an amalgam of declarative (recalling the password or pattern) and procedural (the frequently used motor sequence) memories. (c) App locating speed: This parameter was inspired by the visual search task based on familiar images (Wang et al., 1994). Essentially, the time to perform this task is dictated by both visual attention and memory. This measure is related to visual reaction times (Akeret et al., 2020).

Estimating the periodogram and the corresponding metrics

Lomb-Scargle periodograms were estimated (MATLAB, Mathworks, Natick, USA) and the power was scaled by the input variance. The periodogram was estimated between 0.05 and 12 cycles per day with a step of 0.001 cycles. The statistical significance ($\alpha = 0.001$) of the power fluctuations were estimated against 0 using t-tests (LIMO EEG(Pernet et al., 2011)) and multiple comparisons corrected using the false discovery rate (FDR, also on LIMO EEG). Inputs spanning longer than 10 days were used for this analysis. To compare the periodogram peaks at ~1 cycle per day across the different smartphone and wearable parameters, the peak was determined within the range of 0.7 and 1.6 cycles per day. First, the peaks from the different measures were compared using one-way ANOVA (MATLAB, MathWorks, Natick, USA). These were followed-up with t-tests comparing all possible pairs

of measures. The tests were corrected using Bonferroni correction of Family-Wise Error Rate (FWER, $\alpha = 0.05$, Victor Martinez's Multiple Testing Toolbox as implemented MATLAB)(Benjamini and Yekutieli, 2001). The 95% confidence intervals were estimated using the inverse of Student's T cumulative distribution function (MATLAB). Follow up t-tests after ANOVA to compare the periodogram peaks (location and amplitude) across the different parameters were also corrected using FWER. Inputs spanning longer than 7 days were used for this analysis block focused on ~1 cycle per day rhythm.

Finding signal peak in terms of time-of-the-day using cosinor analysis

The signals were organised as follows: for smartphone usage, luminescence and accelerations, the higher the signal amplitude the more positive the signal. For smartphone tapping speed, unlocking speed and app locating speed, the shorter the inter-touch interval the more positive the signal. The acrophase of the sine wave fits obtained using Cosinor.m (implemented in MATLAB by Casey Cox)(Nelson et al., 1979). Inputs spanning longer than 7 days were used for this analysis. The time-of-the day fluctuations were compared across the different parameters using the Parametric Watson-Williams multi-sample test (Circular Statistics Toolbox for MATLAB) and as a follow-up, the same test was used in pairs. In the paired comparison between peak and off-peak signals, the peak was defined by the cosinor acrophase and the off peak was defined by the cosinor bathyphase. The inter-individual differences in the acrophase across the different parameters were tested for correlation using circular correlation (Circular Statistics Toolbox for MATLAB) (Berens, 2009). The statistical output was corrected for multiple comparisons using the Bonferroni correction of Family-Wise Error Rate (FWER, $\alpha = 0.05$). The 95.0% confidence intervals were estimated using the same toolbox.

In addition, a cosinor independent analysis was used to estimate the time-of-the-day effects on the measured signals. First, the measured signals were binned according to the time-of-the-day at the resolution of an hour. Second, if there were a minimum of 7 samples in each hour, the central tendency was estimated for each bin (mean for luminescence, physical activity, and phone usage, median for TS, US, and ALS; note mean was used due to the presence of '0' values at certain times of the day resulting in sharp edges when using median). This resulted in 24 values for each subject. Subjects where the sample number threshold (of 7) was not reached in > 0 bin were eliminated. Third, the 24 bins were sorted according to signal strength, and the time-of-the-day index was noted for the top 5 bins. These bins were then split into 2 clusters using agglomerative clustering (Circular Statistics Toolbox). The highest-ranking bin of the larger of the two clusters was used to locate the bin with peak performance. The difference between the smartphone parameters was subsequently tested using the Watson-Williams multi-sample test ($\alpha = 0.05$).

Finding signal peak in terms of day-of-the-week

The hourly smartphone and wearable parameters as described above were sorted according to the day of the week. Inputs spanning longer than 10 days were used for this analysis. The mean value from each day of the week was used to derive the location of the peak. These locations were converted into radians towards circular mean and confidence intervals (95%). The measures were compared for day-of-the-week differences across the different parameters as stated above for time-of-the day analysis, that is by using the Parametric Watson-Williams multi-sample test and follow-up paired tests were corrected for FWER.

Estimating performance surrounding sleep

The hourly smartphone parameters were time-locked to the sleep times estimated using the Cole-Kripke algorithm on the actigraphy measures from the left wrist (Cole et al., 1992). The median values in the hour bin preceding, during and after the sleep period was estimated from each individual. To address if there were differences between these three measures were contrasted using two-way ANOVA (MATLAB, MathWorks, Natick, and $\alpha = 0.05$).

Supplementary Figures

Supplementary Figure 1. Related to Figure 2. Absence of rhythms with 90 min period in the proxy measures of cognitive performance captured on the smartphone. Mean periodogram and their corresponding confidence intervals (95%), with the 90 min period marked with a dashed line. Note, no periodogram peak was visible at that period.

Supplementary Figure 2. Related to Figure 3. Smartphone and wearable measures analysed according to the time of the day. (a) The central tendency performance fluctuations for all the participants meeting the sample density requirements to extract signal peak. The signals are normalised at the level of each participant (across rows). (b) The analysis used to identify the peak signal time bins. Note, the clustering method avoids isolated signal peaks. (c) The mean time to peak across the different parameters and the corresponding confidence intervals.

Supplementary Figure 3. Related to Figure 3. Day of the week reflects on physical activity and processing speeds captured on the smartphone. (a) Mean values and the corresponding confidence intervals (95%). (b) The peak performance in terms of the best (mean) performing day of the week and corresponding confidence intervals.

Supplementary Figure 4. Related to Figure 4. Cognitive processing speed captured on the smartphone during actigraphy labelled sleep. Figure legends same as in Figure 4., but the data was separated according to the days of the week of the actigraphy labelled sleep onset.

Supplementary Figure 5. Related to Figure 1. The actigraphy luminescence sensor output when a subject uses the smartphone in a dark room. Light bulb (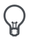) marks the periods when the room was lit.

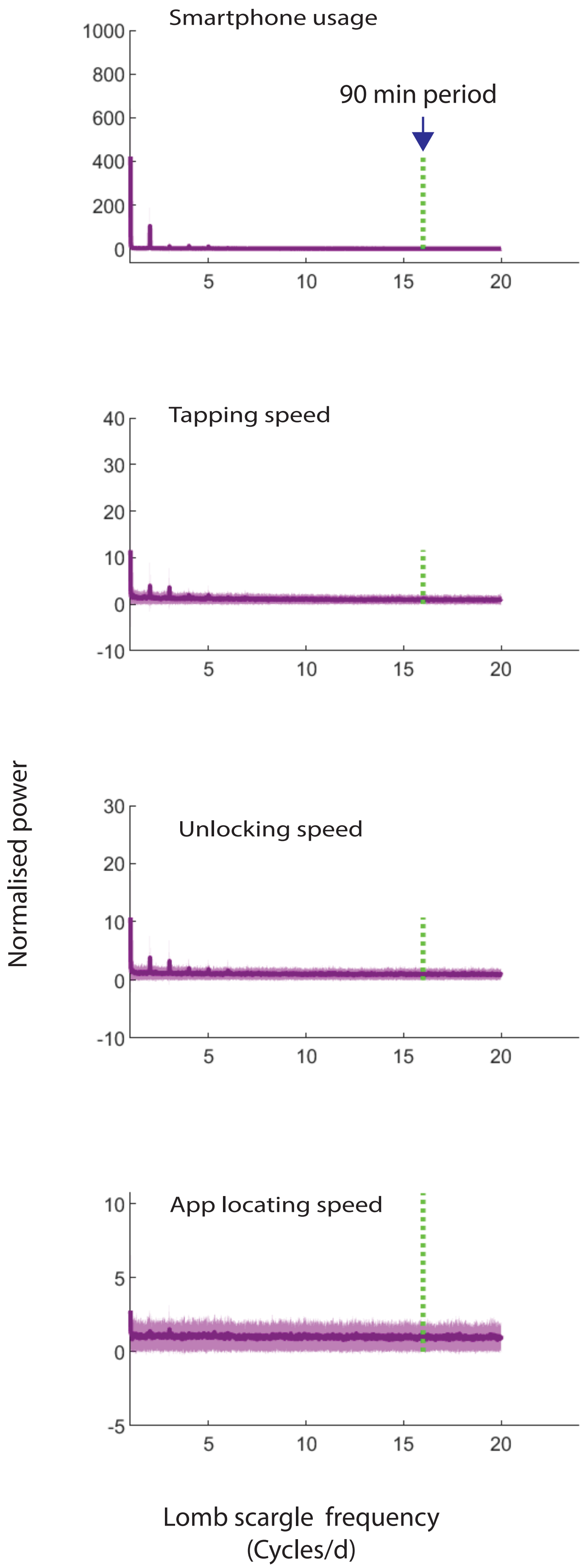
Smartphone usage
90 min period
Tapping speed
Unlocking speed
App locating speed
Normalised power
Lomb scargle frequency
(Cycles/d)

Suppl. Fig. 1

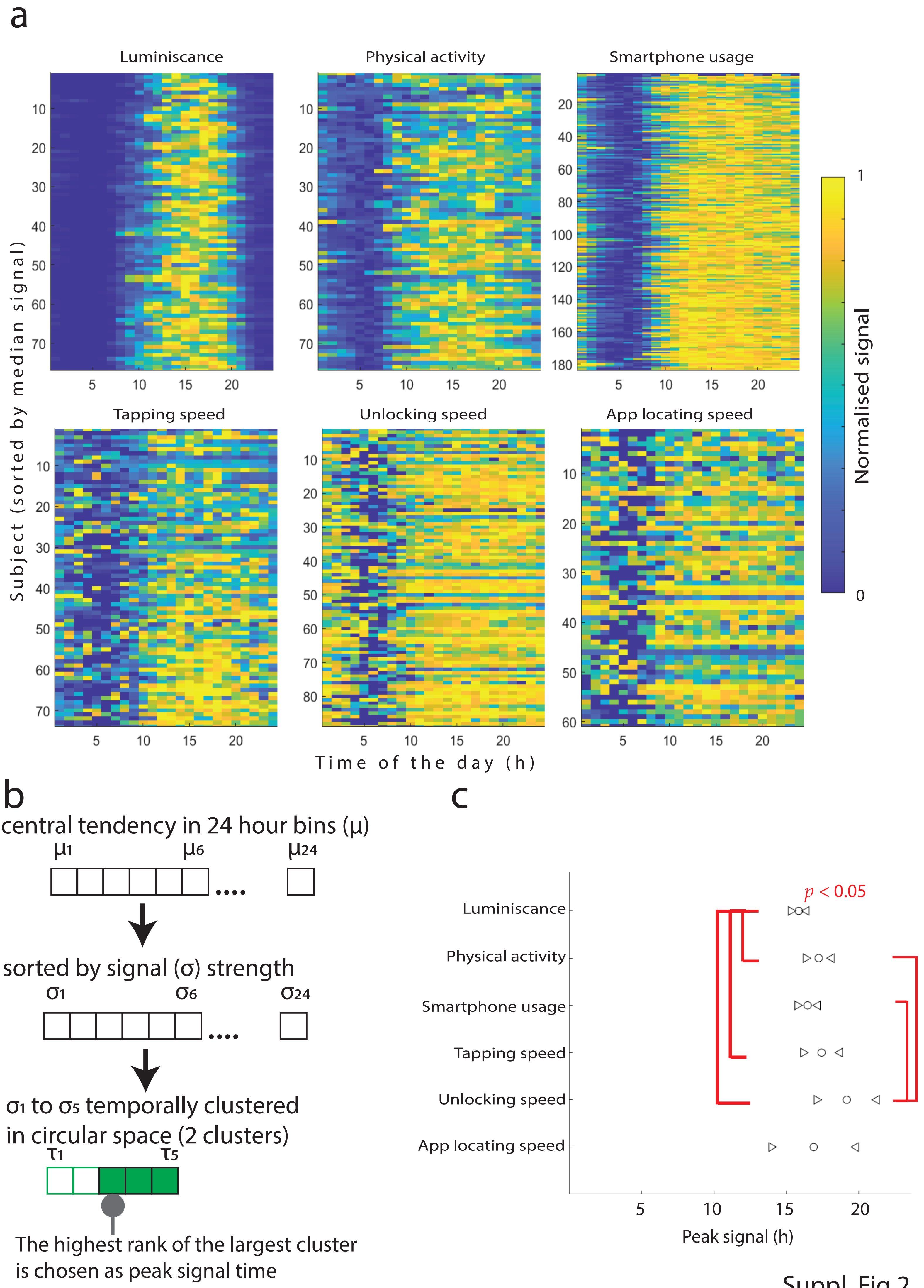
a
Luminiscance
Physical activity
Smartphone usage
Tapping speed
Unlocking speed
App locating speed
Subject (sorted by median signal)
Time of the day (h)
Normalised signal
b
central tendency in 24 hour bins (μ)
μ1
μ6
μ24
sorted by signal (σ) strength
σ1
σ6
σ24
σ1 to σ5 temporally clustered in circular space (2 clusters)
τ1
τ5
The highest rank of the largest cluster is chosen as peak signal time
c
p < 0.05
Luminiscance
Physical activity
Smartphone usage
Tapping speed
Unlocking speed
App locating speed
Peak signal (h)

Suppl. Fig 2.

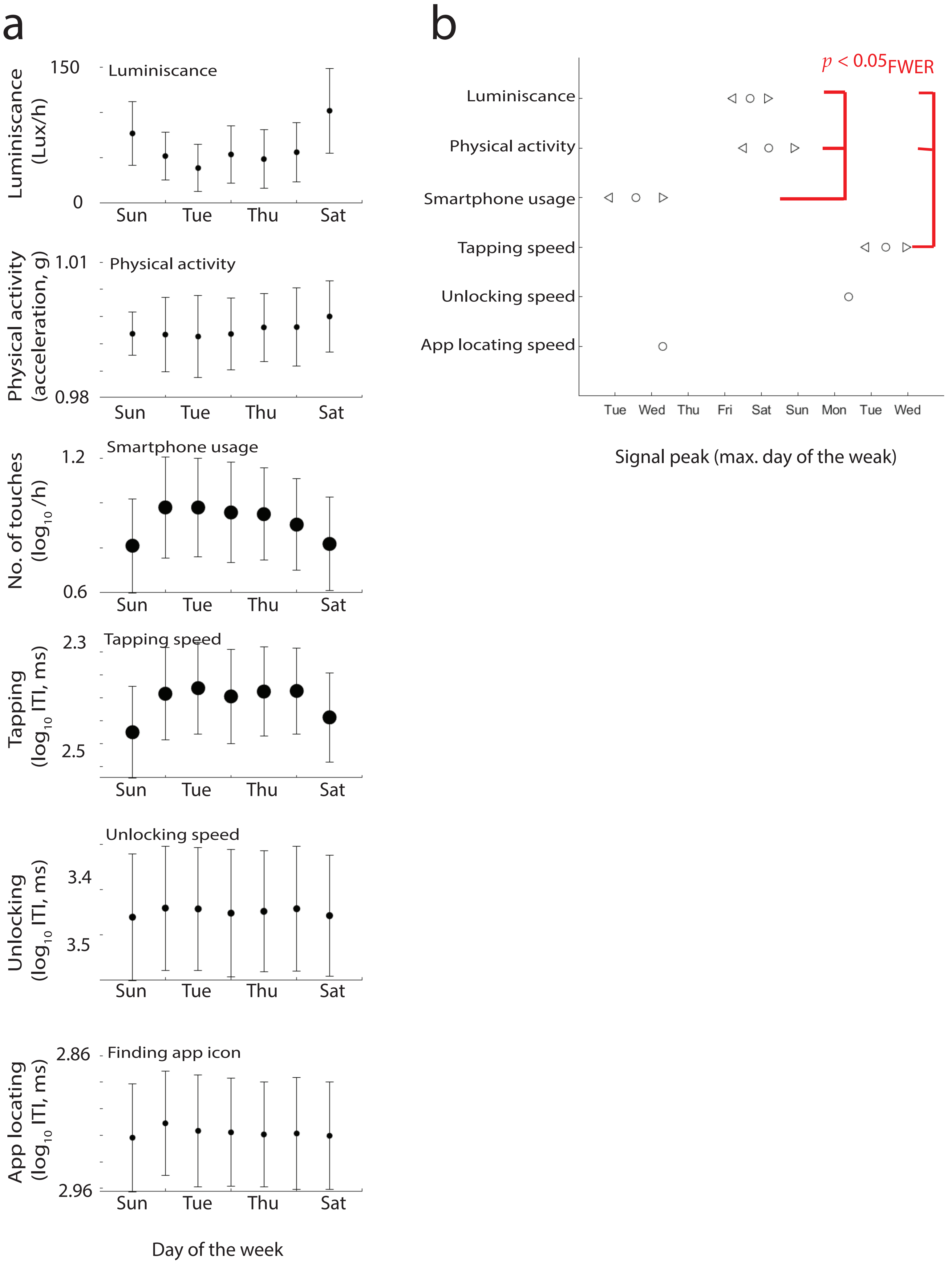
a
Luminiscance
Luminiscance (Lux/h)
150
0
Physical activity
Physical activity (acceleration, g)
1.01
0.98
Smartphone usage
No. of touches (log_{10} /h)
1.2
0.6
Tapping speed
Tapping (log_{10} ITI, ms)
2.3
2.5
Unlocking speed
Unlocking (log_{10} ITI, ms)
3.4
3.5
Finding app icon
App locating (log_{10} ITI, ms)
2.86
2.96
Sun
Tue
Thu
Sat
Day of the week
b
$p < 0.05_{FWER}$
Luminiscance
Physical activity
Smartphone usage
Tapping speed
Unlocking speed
App locating speed
Tue
Wed
Thu
Fri
Sat
Sun
Mon
Tue
Wed
Signal peak (max. day of the weak)

Suppl. Fig. 3

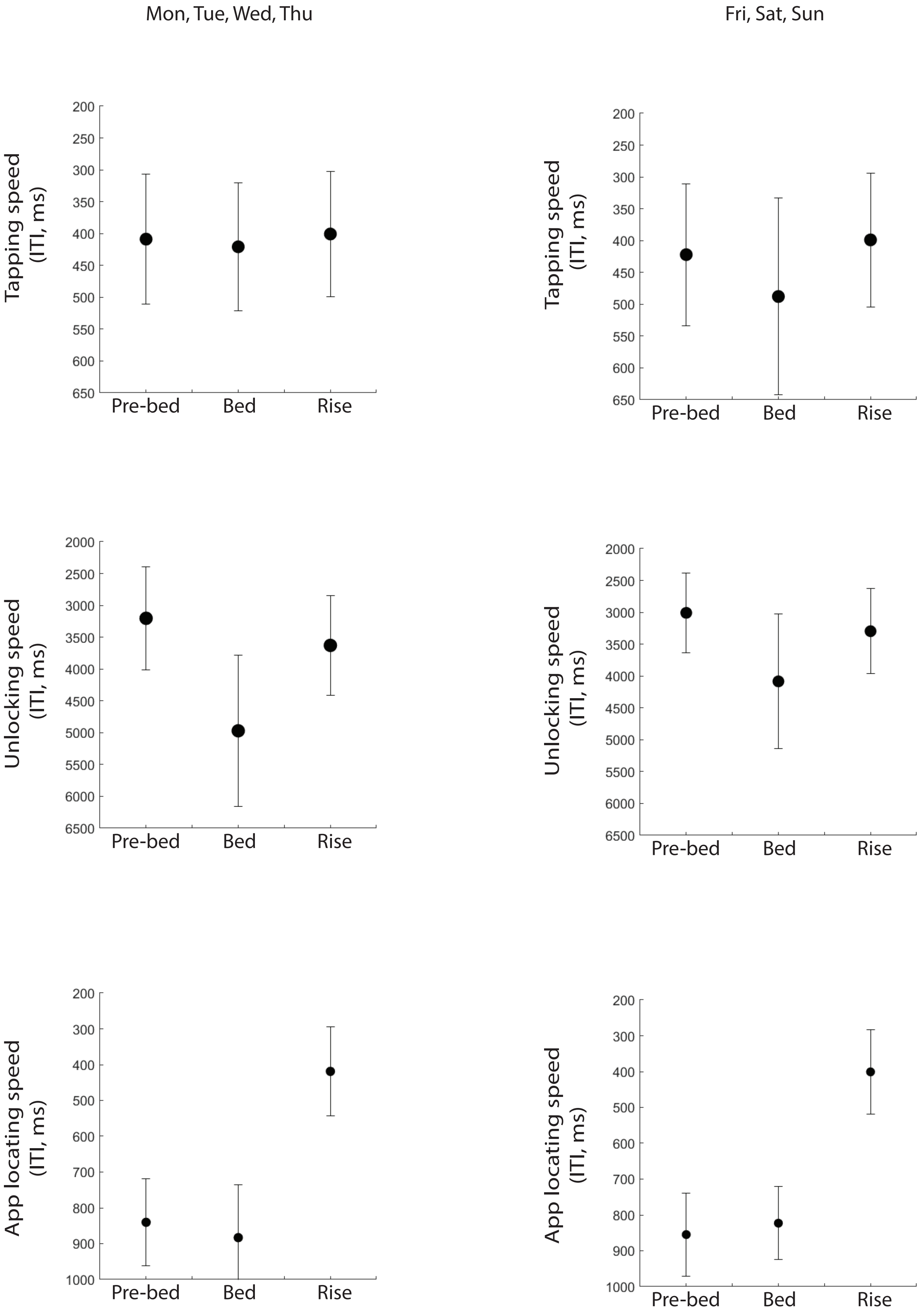
Mon, Tue, Wed, Thu
Fri, Sat, Sun
Tapping speed (ITI, ms)
Unlocking speed (ITI, ms)
App locating speed (ITI, ms)
Pre-bed
Bed
Rise

Suppl. Fig. 4

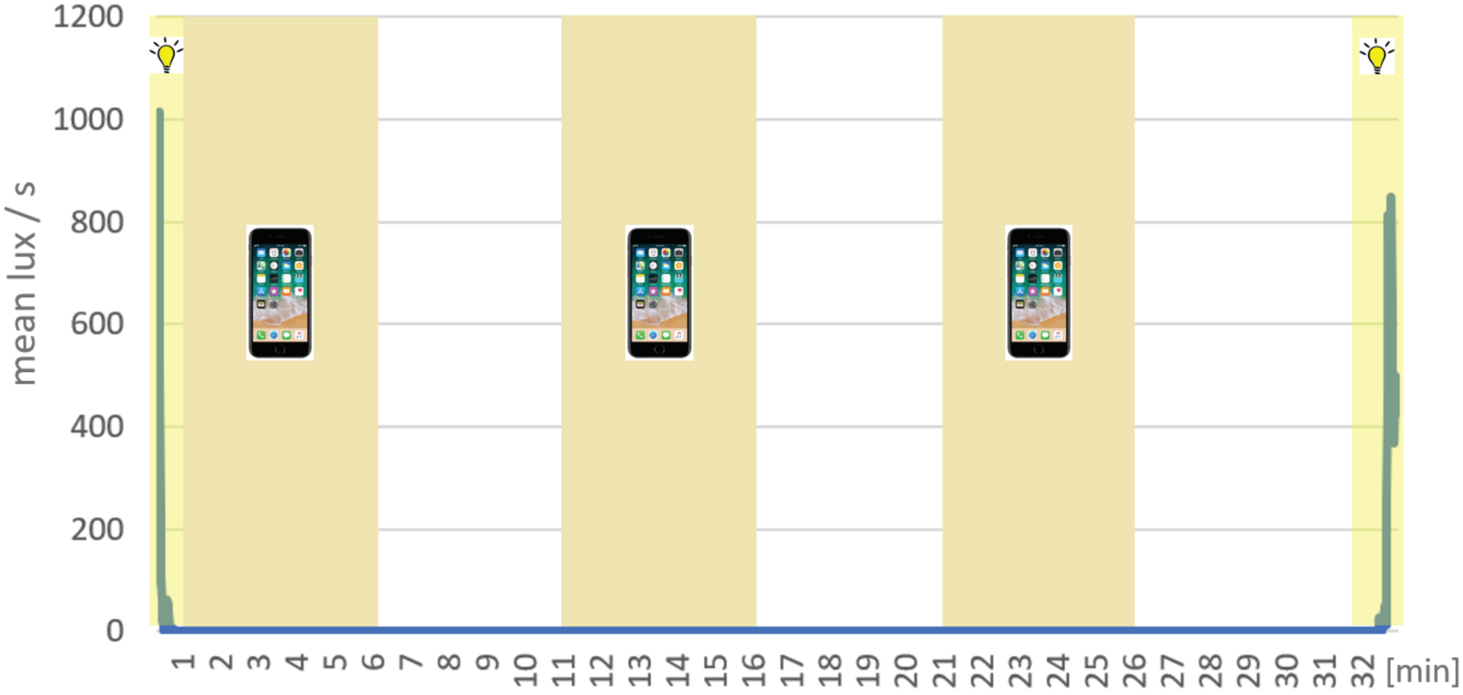

1200
1000
800
600
400
200
0
mean lux / s
1 2 3 4 5 6 7 8 9 10 11 12 13 14 15 16 17 18 19 20 21 22 23 24 25 26 27 28 29 30 31 32 [min]

Suppl. Fig. 5
